# COVID-19 vulnerability: the potential impact of genetic susceptibility and airborne transmission

**DOI:** 10.1186/s40246-020-00267-3

**Published:** 2020-05-12

**Authors:** Krystal J. Godri Pollitt, Jordan Peccia, Albert I. Ko, Naftali Kaminski, Charles S. Dela Cruz, Daniel W. Nebert, Juergen K.V. Reichardt, David C. Thompson, Vasilis Vasiliou

**Affiliations:** 1 Department of Environmental Health Sciences, School of Public Health, Yale University, New Haven, CT 06510 USA; 2 Department of Chemical & Environmental Engineering, School of Engineering & Applied Science, Yale University, New Haven, CT 06520 USA; 3 Department of Epidemiology of Microbial Diseases, School of Public Health, Yale University, New Haven, CT 06510 USA; 4 Section of Pulmonary, Critical Care and Sleep Medicine, School of Medicine, Yale University, New Haven, CT 06520 USA; 5 Department of Environmental Health and Center for Environmental Genetics, University Cincinnati Medical Center, Cincinnati, OH 45267 USA; 6 Australian Institute of Tropical Health and Medicine, James Cook University, Smithfield, QLD Australia; 7 Department of Clinical Pharmacy, Skaggs School of Pharmacy & Pharmaceutical Sciences, University of Colorado Denver, Aurora, CO 80045 USA

## Abstract

The recent coronavirus disease (COVID-19), caused by SARS-CoV-2, is inarguably the most challenging coronavirus outbreak relative to the previous outbreaks involving SARS-CoV and MERS-CoV. With the number of COVID-19 cases now exceeding 2 million worldwide, it is apparent that (i) transmission of SARS-CoV-2 is very high and (ii) there are large variations in disease severity, one component of which may be genetic variability in the response to the virus. Controlling current rates of infection and combating future waves require a better understanding of the routes of exposure to SARS-CoV-2 and the underlying genomic susceptibility to this disease. In this mini-review, we highlight possible genetic determinants of COVID-19 and the contribution of aerosol exposure as a potentially important transmission route of SARS-CoV-2.

## Genomics of susceptibility and resistance

How individuals respond to SARS-CoV-2 exposure is becoming better understood in a global sense, but differences in the vulnerability of individuals to infection and in the spectrum of COVID-19 symptoms remain to be understood. It is known that advanced age and pre-existing conditions (e.g., cardiovascular, pulmonary, and renal diseases) render a person more vulnerable to the more severe health consequences of COVID-19 [1]. However, a surprising observation emanating from the pandemic is the rate of hospitalization of younger, ostensibly healthy individuals.

What makes some people more vulnerable than others to SARS-CoV-2? What role do gene networks play in determining or influencing efficiency of infection, the immune response to infection, or the severity of COVID-19 symptoms? For example, genetic polymorphisms exist in the *ACE2* gene [2], which encodes the cellular receptor for SARS-CoV-2; allelic variants of the *ACE2* may influence the protein’s binding with the virus [3] and subsequent invasion of the cell. In addition, polymorphisms of cellular proteases—believed to facilitate the entry of SARS-CoV-2 into the cell, along with furin [4] and TMPRSS2 [5]—have been shown to exist [6, 7]. Indeed, a recent preprint suggests that *TMPRSS2* variants and resulting expression may influence COVID-19 severity [8]. It is now evident that not all infected patients develop a severe respiratory illness; the reason for this is currently not clear. Moreover, very little is understood about interindividual genetic differences in the immune response to this new and novel version of the old coronavirus. A possible association between the genetic variability in histocompatibility complex (MHC) class I genes (human leukocyte antigen [HLA] A, B, and C) and the susceptibility to SARS-CoV-2 and severity of COVID-19 has recently been suggested [9]. Specifically, the HLA-B*46:01 gene product is predicted to exhibit the lowest binding capacity to SARS-CoV-2 peptides, suggesting individuals with this allele may be more vulnerable to COVID-19—due to reduced capacity for viral antigen presentation to immune cells. Conversely, the authors identified that the HLA-B*15:03-encoded protein is predicted to have the greatest capacity to present highly conserved SARS-CoV-2 peptides that are shared among common human coronaviruses [9]—suggesting patients possessing this HLA genotype may be more likely to develop immunity. Finally, during some viral infections (including HIV), the ADF/cofilin complex (ADF, actin-depolymerizing factor, is encoded by the *DSTN* gene; cofilin is encoded by *CFL1* and *CFL2* genes) is activated. In the initial stages of viral infection, hyperactivation of cofilin and inefficient actin polymerization is known to occur [10]. The possible implication of allelic variants in the *DSTN*, *CFL1*, and *CFL2* genes, as well as the *ACE2* gene, with the spectrum of clinical phenotypes of COVID-19, is therefore intriguing and warrants further exploration.

What makes some SARS-CoV-2 infected individuals extremely sensitive to the development of acute respiratory distress syndrome (ARDS) while others are asymptomatic. In this day and age of next-generation sequencing, perhaps groups of 500 or 1000 age- and gender-matched patients could have their entire genomes sequenced, and then sophisticated genetic analysis software programs could home in on single-nucleotide variant (SNV) differences in possibly relevant genes or genomic regions in the afflicted (highly sensitive) and the asymptomatic (highly-resistant) groups.

Our understanding of genetic susceptibility to SARS-CoV-2 infection and the severity of COVID-19 is still in its infancy. The scientific community is trying to address this issue by combining research efforts using existing genetic databases. An important step in this direction is the COVID-19 Host Genetics Initiative, created by Mark Daly and Andrea Ganna from the Institute for Molecular Medicine Finland (FIMM); this initiative is intended to encourage the human genetics community to generate, share, and analyze data to elucidate the genetic determinants of COVID-19 susceptibility, severity, and outcomes (https://www.covid19hg.org/). So far, this initiative shows promise because major biobanks (e.g., FinnGen) have expressed a willingness to participate [11]. In addition to this initiative, the UK Biobank—which contains samples from 500,000 volunteers and detailed information about their health—has now started to curate data from COVID-19 patients (https://www.ukbiobank.ac.uk/2020/04/covid/).

In Iceland, the company deCODE Genetics has monitored the spread of the virus using SARS-CoV-2 genomic analysis [12] and has partnered with Iceland’s government to sequence the genomes of viral hosts, i.e., patients who were previously infected with COVID-19 (https://www.decode.com/). The Greek government has recently funded an initiative, COVID-19-GR, to genotype 3500 COVID-19 patients, whole-genome sequence their SARS-CoV-2 genome, and perform immunogenomic analyses. All of these data will be linked with detailed clinical information collected in the Greek COVID-19 registry (http://www.gsrt.gr/central.aspx?sId=119I428I1089I646I488772). In the USA, Harvard’s Wyss Institute researchers and the Personal Genome Project at Harvard University are launching a project to compare the genomes, microbiomes, viromes, and immune systems of consenting individuals with extreme COVID-19 susceptibility and individuals that exhibit resistance (https://wyss.harvard.edu). Finally, the Yale SARS-CoV-2 Genomic Surveillance Initiative is sequencing the genome of SARS-CoV-2 in order to monitor the spreading of the virus in Connecticut (https://covidtrackerct.com/).

In addition to the genomics research, attention needs to be paid to the spread of the virus and how it can be prevented. Below, we therefore review the latest on transmission of SARS-CoV-2.

## Understanding the impact of airborne coronavirus on transmission

The transmission efficiency of SARS-CoV-2 has proved to be high, with reported reproductive numbers greater than that of the 2009 H1N1 influenza virus [13, 14]. The routes of exposure that have led to this high transmissivity have been the subject of considerable discussion, notably the contribution of aerosol transmission. As with any infectious respiratory disease, an infected individual can release aerosols and droplets containing SARS-CoV-2 by coughing or sneezing [15–18]. Similar to what we know about influenza A and B, MERS-CoV, and SARS-CoV-1, these virus-containing aerosols and droplets can lead to short-range airborne transmission (~ 6 ft) [19, 20]. Such aerosols (< 10-μm diameter) and droplets (> 10-μm diameter) can promote infection through (i) deposition on surfaces [21] and subsequent hand-to-mouth/nose/eye transfer and (ii) inhalation. While suspended airborne droplets can persist in the air for several minutes, the smaller aerosols do not rapidly settle and can persist for longer durations (~ minutes to hours) [22]. Once airborne, the characteristics of aerosols generated by cough or sneeze are dynamic, notably decreasing in size due to evaporative loss of water depending on ambient humidity and temperature levels [23, 24]. As the size of aerosols decrease, their ability to disperse in the air is enhanced. Therefore, inhalation of aerosol-borne SARS-CoV-2 is likely to be a relevant mode of viral infection, with the range of aerosol transmission extending beyond 6 ft of an infected individual.

## Routes of aerosol generation

Beyond coughing and sneezing, normal speech and breathing can also generate aerosol. The size of aerosol generated by speaking and breathing is similar, ranging from 0.75 to 1.1 μm, but is notably smaller than those generated by coughing or sneezing, i.e., ~ 5 μm [25]. Prior studies that collected exhaled breath condensate from influenza patients have demonstrated that this respiratory virus could be emitted just by breathing [26]. The small size of aerosols released through this route potentially extends their range of travel. The concentration of aerosol released by the combination of speaking and breathing for more than 4 min is equivalent to the amount of aerosol emitted for 30 s of singing or coughing [15, 27, 28]. The volume of speech can further influence aerosol release, leading to variations in emission rates between individuals that may impact their capacity for viral transmission [25]; this is relevant, in particular, for infected individuals that are pre-symptomatic or have asymptomatic illness. Transmission of aerosol generated through these routes over short distances has been supported by recent case studies of family clusters in various Chinese cities [29, 30], a restaurant setting in Guangzhou, China, [31] and a choir group in Mount Vernon, WA, USA [32]. In addition, droplets or aerosols deposited on floors and surfaces can become resuspended by human activity, and resuspended particles can comprise the majority of the aerosols within an occupied setting [33, 34].

## Cases of airborne SARS-CoV-2 detection

Over the past weeks, three recent studies conducted in Wuhan, China, [35]; Singapore [36]; and Omaha, NE, USA [37], have reported SARS-CoV-2 detection in indoor air samples. These studies sampled air with active samplers that used a pump to draw air through a filter or into a cylindrical chamber where airborne material was collected. However, they all used different sampling systems, which further varied by the sampling flow rates, collection times, and sizes of aerosol sampled. Using quantitative RT-PCR, airborne levels of viral RNA were found in rooms of COVID-19 patients. Virus was detected in the air sampled from the rooms of symptomatic, as well as asymptomatic, patients. Interestingly, levels of airborne SARS-CoV-2 in these patients’ rooms decreased as their illness progressed. In the Wuhan hospital study, the authors suggested that in addition to direct emission, resuspension of viruses from droplets deposited on personal protective equipment (PPE) or flooring was a source of SARS-CoV-2 aerosols [35]. While these initial reports suggest SARS-CoV-2 can be detected in the air, it is important to acknowledge the small sample sizes of these studies, as well as those lacking appropriate controls.

Airborne SARS-CoV-2 RNA was also detected in hallway spaces outside patients’ rooms [37] and in medical staff areas of the hospital, i.e., changing rooms, office spaces, and PPE removal rooms [35]. Concerningly, concentrations measured in the medical staff areas often exceeded those found in the patient rooms. In addition, the virus-containing aerosols in the sampled air of the medical staff areas were smaller in size (peak concentrations appearing in the submicron (0.25 to 1 μm) and supermicron (> 2.5 μm) ranges) than those in patient rooms (1 to 4 μm and > 4 μm [36]). Identifying SARS-CoV-2 in a range of aerosol size fractions raises further questions about the deposition profile of the virus in the lung. It is well established that larger aerosols (> 4 μm) are predominantly deposited in the upper and central airways (i.e., nasopharynx, tracheobronchial) and are subject to mucociliary clearance. In contrast, deposition in the deep lung (i.e., alveoli), having epithelial cells rich in ACE2 [38], is enhanced for smaller aerosols (< 4 μm), thereby potentially facilitating its transmission efficiency.

Some indoor public spaces tested in Wuhan, China, were found to have detectable levels of SARS-CoV-2, including the hospital pharmacy and department stores [35]. Airborne levels of the virus in these public spaces were comparable to concentrations detected in hospital patient workspace areas but lower than measurements in medical staff areas.

## Disease transmission by SARS-CoV-2 aerosols

Detection of SARS-CoV-2 in indoor air of hospitals and public spaces raises several questions: What is the ability for this airborne form of the virus to transmit disease (i.e., is the virus viable)? And how do measured viral concentrations in the air (which, to date, have been only accomplished through PCR-based approaches) relate to an infectious dose?

The premise of aerosol transmission necessitates that the virus remains viable in the air. As viability decreases over time, it is important to understand the rate of inactivation under different environmental conditions, e.g., temperature and humidity [39]. In recent laboratory testing, aerosolized SARS-CoV-2 remained viable for up to ~ 3 h [21] when tested at a relative humidity of 65%. Inactivation studies suggested that survival on surfaces, and in the air, may be further enhanced at relative humidities of less than 50% [40]. These results are consistent with other airborne viruses—including SARS-CoV-1 [21, 41], influenza H1N1 [42, 43], and MERS-CoV [44–46], which also show evidence of airborne transmission. Viability of SARS-CoV-2 in aerosol collected from real-world air samples, however, has not yet been fully explored. Only one of the three studies that detected SARS-CoV-2 in hospital air examined the viability of the virus. No evidence of viral propagation was found for air samples collected at the University of Nebraska Medical Center [37]. This negative result may be attributable to low airborne levels of the virus [47], but it is also feasible that the air sampling methods used may have contributed to reduced viability of the sampled virus. In Nebraska, air samples were collected at a stationary indoor location on gelatin filters at a flow rate of 50 L/min for 15 min. Air sampling parameters, such as flow rate, influence virus detection and viability [47]. It is critical that air sampling methods for infectious virus do not compromise the integrity of the viral envelope because this would reduce or eliminate infectivity and contribute to false negatives.

Detection of SARS-CoV-2 in the air prompts questions about safe exposure levels. The high transmissivity of the virus suggests that a low dose might be sufficient to infect an individual; however, such studies have yet to evaluate the infectious dose of SARS-CoV-2. Until scientific evidence emerges, it is useful for individuals to follow approaches that minimize their risk of infection by reducing their exposure level *and* duration of exposure. Initial studies (as detailed above) report a range of airborne virus exposure levels in hospitals, as well as public spaces. The *combined use* of masks and physical distancing can be effective approaches for decreasing exposure to airborne forms of SARS-CoV-2. Avoiding or minimizing the time in contact with these potential aerosol exposures would also be a critical parameter in lowering risk.

## Preventing new infections

An inherent difficulty associated with the airborne route of transmission is that the measures used to reduce airborne exposure can be laborious and are not fail-proof. Common approaches for mitigating airborne exposures include (i) identification of emission sources, (ii) prevention of viral shedding and inhalation exposure, and (iii) environmental controls. The topic of environmental controls leverages evidence of reduced exposures by improving ventilation [48], utilization of portable filtration devices [49], or other aerosol inactivation technologies, and cleaning practices to reduce exposure from resuspension [50]. The topic of environmental controls is broad and complex. Therefore, we defer to guidance prepared by the American Society of Heating, Refrigerating and Air-Conditioning Engineers (ASRHAE) and other recent papers focused on indoor ventilation and environmental controls [51, 52].

### Identification of emission sources: personal exposure assessment

Viral shedding by an individual has been suggested to be greatest immediately following infection, prior to onset of symptoms [53]. Unfortunately, current testing protocols require most individuals to have developed multiple symptoms of COVID-19 in order to qualify for testing, after which a confirmed diagnosis can be made. Monitoring airborne levels of SARS-CoV-2 may be an alternative method for identifying exposure hotspots and could be used to alert individuals, at increased risk of infection, to practice self-isolation measures. Such exposure testing might be conducted at fixed indoor locations of particular concern, such as sites with vulnerable individuals (e.g., nursing homes) or high activity (e.g., groceries stores). Wearable air sampling equipment is also available and can be used to assess personal SARS-CoV-2 exposure. For example, at the University of Nebraska Medical Center, personal exposure samples were collected from individuals in direct contact with COVID-19 patients (who had experienced no cough at the time of sampling) [37]; the viral levels measured on personal devices were found to be higher than those taken at a stationary site in the patient’s room. It is, however, important to appreciate that only two personal exposure samples were evaluated in this study. Understanding an individual’s exposure to airborne forms of the virus and their potential for infection may circumvent further exposures across a community.

### Prevention of viral shedding and inhalation exposure: effective face coverings

A range of face coverings is available—including N95 respirator masks, surgical masks, and cloth coverings, each offering different efficiencies for inward protection (i.e., PPE) and outward protection (i.e., source control) from virus-laden aerosol and/or droplets. For inward protection of the individuals wearing the masks, the gold-standard N95 masks are capable of filtering > 99% of airborne aerosols; this is in contrast to filtration efficiencies of surgical masks (~ 75%) and cloth coverings (~ 67%) that afford inward protection against for aerosols sized between 0.02 and 1 μm [54, 55]. For outward protection of other individuals from the individual wearing the mask, the N95 has been shown to prevent ~ 70% of aerosols (0.2 to 1 μm) generated by coughing and then from being released. On the other hand, surgical masks and cloth masks leaked ~ 50% and ~ 90%, respectively, of the same emissions [54, 55]. Face coverings have been recommended by many public health agencies internationally with the aim of preventing new infections. Because the supply of surgical and N95 masks are limited such that available resources are prioritized for health care providers, the general public has been recommended to use homemade cloth face covering to mitigate exposure of healthy individuals and prevent release of aerosolized virus shed from infected individuals [56]. With the increased use of cloth masks, it is important to recognize their limitations and to consider design modifications for improvement. Notably, the filtration efficiency (for inward or outward protection) of a cloth face covering varies by the type of material used [57, 58]. Use of a single-ply thin fabric (such as cotton, silk, or linen) has a relatively low capacity for capturing submicron (< 1 μm) virus-laden aerosol. Materials such as vacuum cleaner bags (made of heavy, dense, random-fiber orientation material) have demonstrated a barrier to aerosol transport that is similar to surgical masks. In addition to the choice of material, the loose fit of homemade cloth masks may also contribute to poor filtration efficiency. This issue is most important in children. Ensuring this age group is well protected through proper fit of cloth face coverings is critical given the high prevalence of asymptomatic cases reported in children [59]. While promoting the use of face coverings by the public, it is also essential to ensure cleaning protocols prior to reuse, and to reinforce the importance of continued physical distancing to prevent individuals from having a false sense of security.

## Conclusions

The contribution of aerosol exposure to the transmission of SARS-CoV-2 has been the subject of recent intensive debate. Whereas the World Health Organization has dismissed this mode of transmission [60], scientists have emphasized that infected individuals represent emission sources of aerosol generated by routine behaviors—such as breathing, speaking, singing, coughing, sneezing, and resuspension activity—all of which *might* be capable of transmitting disease [61, 62]. Because the short history of the COVID-19 pandemic has been marred by large amounts of misinformation, it becomes critically important to provide a definitive answer to the question as to whether or not the disease is transmitted by aerosol. This requires the development, standardization, and dissemination of optimal techniques for sampling airborne SARS-CoV-2. Such techniques are essential for the adoption of proven strategies that limit exposure to SARS-CoV-2 and will likely decrease new cases and protect the population, when the pandemic wanes and return-to-work strategies are being implemented.

The possibility of a second wave of SARS-CoV-2 infections is very real; hence, preventive measures are important. It is noteworthy that vaccines against respiratory syncytial virus (RSV), rhinoviruses, SARS-CoV-1, and MERS-CoV have not yet been successful. Thus, for SARS-CoV-2 (and these other viruses), the medical and scientific communities must intensify their studies in the areas of drug development for discovery of efficacious therapies and in preventive measures. Prevention would include avoidance of viral contamination, as well as possible identification of *genetically susceptible subgroups* within the human population.

Looking forward and in the context of this journal, the genomics of susceptibility to SARS-CoV-2 infection, as well as the wide variation in clinical response to COVID-19 in patients, should become active areas of investigation. Variations in COVID-19 severity might be classified as (a) asymptomatic, (b) symptomatic but no hospitalization required, and (c) severely symptomatic with hospitalization urgently indicated. Elucidation of alleles of relevant genes associated with these three levels of severity to viral response might aid clinicians in dealing with possible future waves of this pandemic. Elucidation of genomics and genetic pathways related to susceptibility of SARS-Cov-2 infection could also become important in combating a future wave. Results from such studies are therefore of great interest to this journal; therefore, we at *Human Genomics* strongly encourage future submissions in these areas of genomics and genetic pathways.

## Data Availability

Not applicable
